# Does economic inequality moderate the effect of class on prosocial behavior? A large-scale test of a recent hypothesis by Côté et al

**DOI:** 10.1371/journal.pone.0220723

**Published:** 2019-08-09

**Authors:** Hagen von Hermanni, Andreas Tutić

**Affiliations:** Institut für Soziologie, Universität Leipzig, Leipzig, Germany; Rice University, UNITED STATES

## Abstract

Empirical research has provided mixed evidence regarding the question of whether higher social class promotes prosocial behavior. Recently, Côté et al. [1] hypothesized that these conflicting evidences might result from a hitherto neglected interaction between the individual’s level of income and the degree of inequality in one’s society. They argue that societies with a higher level of inequality foster a sense of entitlement in high-income individuals, which in turn leads them to be less generous. We put this reasoning to a large-scale test using observational data from the European Social Survey (ESS) and push the scope of our investigation towards a broader conception of social class, using next to income two additional measures of class. First, we examine whether high-class individuals in societies with high levels of inequality do in fact feel more entitled than their counterparts in more equal societies. While we find that an individual’s class and the disposition towards entitlement are strongly correlated, our results show a negative interaction with inequality, i.e. the effect of class on the personal sense of entitlement is weaker in societies with high levels of inequality. Second, we test whether the effect of class on prosocial behavior is moderated by economic inequality with respect to two real-life acts of prosocial behavior, namely engaging in volunteer work and donating money to a humanitarian organization. Our results indicate a substantial positive effect of class on prosocial behavior throughout, as well as a moderate, yet positive, interaction effect of class and inequality.

## Introduction

Since 2010 a number of psychological and sociological studies have been published, which deal with the question of whether socioeconomic classes differ regarding prosocial behavior. Most notably, Piff and colleagues have argued that high-class actors generally tend to act less prosocial and more unethical than low-class actors [2,3]. They supported this claim by a number of experimental studies with subjects from the US using income, education or a subjective measure of social status as proxies for social class. For instance, they demonstrated that high-class subjects donate less to an anonymous stranger in the dictator game and drive more recklessly than subjects of lower class.

At the same time, scholars from the EU published results which point towards the contradicting claim, i.e. they demonstrated that high-class actors behave more prosocial than low-class actors [4–6]. For example, ninth-graders attending better schools in the highly-stratified German educational system donate higher amounts to anonymous strangers in the dictator game than ninth-graders from worse schools [5]. Similarly, medical doctors donate higher amounts than nurses and nursing students in hospitals [6]. Using large-scale sociological data sets, it has been demonstrated that high-class subjects donate higher percentages of their income to charitable organizations and have a higher propensity to engage in volunteer work than low-class subjects [4].

Against this background, Côté et al. reasoned that the conflicting evidences between studies from the US and the EU might be caused by a moderation of the effect of income on prosocial behavior by economic inequality [1]. More specifically, it is argued that there is a negative interaction between inequality and income. Under conditions of severe inequality, entitlement might spread among high-income actors which makes them less inclined to act prosocial and cause a negative correlation between income and prosocial behavior. Given rather mild economic inequality, however, this negative relationship between income and prosocial behavior might weaken and possibly even reverse. The authors present evidence from two studies with 1500 and 700 subjects from the US respectively. Since then this hypothesis has been integrated into the so-called ‘Inequality maintenance model of social class’ [7], which describes several mechanisms by which high- and low-class actors reinforce instead of mitigate social inequality.

We expand on the ongoing discourse by testing Côté et al.’s theoretical reasoning in three novel ways: First, while Côté et al. limit their hypothesis regarding the moderating effect of inequality to one particular instance of class, i.e. income, we test its validity with respect to three different measures of social class. Besides income per se, we also employ a composite of education, income, and occupational status as well as subjective social status [8]. Working with multiple and in particular broader measures of social class strengthens the relevance of Côté et al.’s intriguing hypothesis for the aforementioned strand of literature on the relationship between social class and prosociality. Moreover, it provides additional robustness for our findings. Second, since the ESS contains a standard measure for the psychological concept of human values, we can test the theoretical mechanism underlying Côté et al.’s hypothesis. That is, we check empirically whether the degree of inequality leads individuals of higher social class to feel more entitled. Third, using observational data from the European Social Survey (ESS) allows us to consider the joint impact of income inequality and social class across several heterogeneous societies on acts of real-life generosity, namely the donation of money and time (in the form of voluntary work) to charitable organizations. Notably, income inequality as measured by the Gini coefficient varies more widely in our European sample than in Côté et al.’s initial studies.

## First inquiry–entitlement

In this section we test whether the effect of social class on entitlement is moderated by economic inequality. Before we delve into the actual analyses, we briefly provide the methodical underpinnings of all of our three inquiries (for further details see Measures).

The ESS is a cross-national survey conducted biennially since 2002 comprising a varying number of member states of the EU [9]. The ESS datasets contain paradata that allows to identify the respondents dwelling on a subnational level. These regional identifiers correspond (mostly) with the *Nomenclature of Territorial Units for Statistics* (NUTS)–a standard regulated by the EU, which divides countries artificially into regions with roughly equal populations for administrative and statistical reasons and is used by Eurostat and the OECD [10,11]. The nomenclature allows the distinction of four levels, of which the largest three are used in our analyses: NUTS-0 corresponds to the country itself, NUTS-1 to regions with more than 3 million inhabitants, and NUTS-2 to regions with between 800000 and 3 million inhabitants. This allows us to obtain a recent measure of regional income inequality as well as corresponding values for the state-level variables used in Côté et al.’s studies.

Part of the ESS core questionnaire is a multidimensional measure of human values as proposed by Schwartz [12]. This allows us to use the higher-order human value of ‘self-enhancement’ (comprising the lower order values of ‘power’ and ‘achievement’) as a proxy for psychological entitlement, describing the extent to which people are willing to ‘enhance their own personal interest even at the expanse of others’ [13,14]. Using data from the sixth wave of the ESS from 2012, we construct an indicator for entitlement by principal component analysis (PCA). This indicator is higher for individuals who deem it important to receive respect from others, their orders to be followed, want to be recognized for their achievements, and want to be rich and own expensive things. In the following we will refer to this indicator as entitlement.

While on a conceptual level self-enhancement and entitlement differ, taking the former as a proxy for the latter can be justified as follows: Côté et al. assume that under conditions of high levels of inequality high-income actors engage in ‘favorable downward social comparisons’ which lead them to ‘acquire a sense of entitlement, the belief that one is more important and deserving than others […] and that resources rightfully belong to them’. These assumptions are in line with a wider field of research, which has not just identified a negative association between social class and the propensity of prosocial behavior, but also its mediation by personal traits characterizing narcissistic personalities, like entitlement, grandiosity and high-levels of self-interest [15,7]. These findings are highly plausible, considering that narcissism and especially the Entitlement/Exploitativeness (E/E) subscale, as measured by the Narcissistic Personality Inventory (NPI), have been shown to be strongly ‘associated with promoting the self at the expanse of others’ [14,16]. In addition, even if there remain some doubts regarding the suitability of self-enhancement as a proxy for entitlement, our analyses remain relevant for research on the interplay of social class and prosocial behavior, because self-enhancement can be considered as some form of a prosocial orientation.As a measure for the respondents’ social class we build a composite equaling the standardized average of respondents’ education (ISCED), occupational prestige (SIOPS), and the household equivalized income, converted to a purchasing power parity standard in US dollars [17]. In addition to the standard ESS questionnaire, the sixth wave also contains a measure of the subjective socioeconomic status, asking respondents to identify their ‘place in society’ on an eleven-tier scale [18].

After aligning the ESS regional identifier with the NUTS nomenclature, and after case-wise deletion of all observations containing missing values, we are able to identify 127 regions in 16 countries with an overall sample size of 20715 subjects. As a first step of our analyses, we check whether our measures of social class do show a positive association with levels of entitlement, as has been proposed by social psychologists [15]. As can be seen in Table 1 our findings corroborate a positive relationship: Individuals who place themselves on standard deviation higher in society do also show significantly higher levels of entitlement (0.17, p<0.000).

**Table 1:** Regression coefficients (βs) for the multilevel models regressing entitlement on class measures.

	Subjective status		Equivalized income (PPP)		Objective class	
	β	SE	β	SE	β	SE
Subjective status	0.17***	(0.01)				
Equivalized income (PPP)			0.07***	(0.01)		
Objective class					0.08***	(0.01)
n	20715		20715		20715	

All variables are globally z-standardized (M = 0, SD = 1).

* p < 0.05

** p < 0.01

*** p < 0.001

Next, in order to test Côté et al.’s assumption as comprehensive as possible, we calculate three separate multilevel regression models (using Stata’s mixed-ado) which each regresses our entitlement variable on one of three class measures (subjective status, income, and objective class), the Gini coefficient for income inequality before taxes and social transfers as a second-level predictor, as well as their respective cross-level interactions.

Following Côté et al.’s reasoning we would expect to find (at least) two positive effects in each regression model–a positive association between class (however it is measured) as well as a positive interaction between class and inequality. As we can see in Table 2, all three class measures show in fact a positive effect on the individual level of entitlement. Individuals who consider themselves to stand higher in society’s hierarchy, have a higher equivalized income or individuals with higher social class (as measured jointly by income, education and prestige) do feel on average more entitled. On the other hand, local levels of inequality do not directly influence the distribution of this disposition across regions. Finally, we do find a moderation in two out of three models, although in the form of a negative interaction. While there is no interaction between income and inequality, people of higher subjective status and objective social class seem to be more modest where inequality is particularly pronounced.

**Table 2:** Regression coefficients (βs) for the multilevel models regressing entitlement on class measures and regional levels of inequality.

	Subjective status		Equivalized income (PPP)		Objective class	
	β	SE	β	SE	β	SE
Inequality	0.02	(0.04)	0.01	(0.04)	0.01	(0.04)
Subjective status	0.17***	(0.01)				
Subjective status * Inequality	-0.02**	(0.01)				
Equivalized income (PPP)			0.07***	(0.01)		
Equivalized income (PPP) * Inequality			-0.01	(0.01)		
Objective class					0.08***	(0.01)
Objective class * Inequality					-0.03***	(0.01)
n	20715		20715		20715	

* p < 0.05

** p < 0.01

*** p < 0.001

As a robustness check and for comparison with the results of Côté et al. we repeat our analyses including further individual (age, sex, minority, employment status, marital status, religiosity, conservative ideology) as well as regional- or state-level predictors (average income, population size, percentage urban, age diversity, sex diversity, origin diversity) that could correlate with inequality as well as the dependent variable. As can be seen in Table 3, the full models support our results and show also a significant negative effect in case of the interaction between inequality and income.

**Table 3:** Regression coefficients (βs) for the multilevel models regressing entitlement on class measures and regional levels of inequality–controlling for additional individual- and regional-level variables.

	Subjective status		Equivalized income (PPP)		Objective class	
	β	SE	β	SE	β	SE
Inequality	0.02	(0.03)	0.01	(0.03)	0.01	(0.03)
Subjective status	0.16***	(0.01)				
Subjective status * Inequality	-0.02*	(0.01)				
Equivalized income (PPP)			0.07***	(0.01)		
Equivalized income (PPP) * Inequality			-0.01*	(0.01)		
Objective class					0.06***	(0.01)
Objective class * Inequality					-0.03***	(0.01)
Age	-0.22***	(0.01)	-0.23***	(0.01)	-0.23***	(0.01)
Gender	-0.24***	(0.01)	-0.24***	(0.01)	-0.24***	(0.01)
Minority	0.26***	(0.03)	0.22***	(0.03)	0.22***	(0.03)
Paid work	0.01	(0.01)	0.01	(0.01)	0.01	(0.01)
Married	-0.03*	(0.01)	-0.00	(0.01)	-0.01	(0.01)
Religiosity	0.01	(0.01)	0.02*	(0.01)	0.02*	(0.01)
Conservatism	0.07***	(0.01)	0.09***	(0.01)	0.09***	(0.01)
Average income (PPP)	-0.07	(0.04)	-0.07	(0.04)	-0.06	(0.04)
Population size	0.06	(0.04)	0.06	(0.05)	0.06	(0.05)
Percentage urban	-0.10*	(0.04)	-0.10*	(0.04)	-0.09*	(0.04)
Age diversity	-0.18***	(0.03)	-0.19***	(0.03)	-0.18***	(0.03)
Sex diversity	-0.14***	(0.03)	-0.12***	(0.03)	-0.12***	(0.03)
Origin diversity	0.00	(0.03)	-0.00	(0.03)	-0.00	(0.03)
n	20715		20715		20715	

Sociodemographic variables are part of the ESS core questionnaire, regional variables retrieved from Eurostat. Age was recoded into 14 five-year groups and z-standardized afterwards. Gender is coded 0 = male and 1 = female. Minority is coded 0 = not belonging to minority group in country and 1 = belonging to minority group. Paid work = 1 if respondent engaged in paid work in the past 7 days, zero otherwise. All other variables are globally z-standardized (M = 0, SD = 1).

* p < 0.05

** p < 0.01

*** p < 0.001

**Fig 1:**
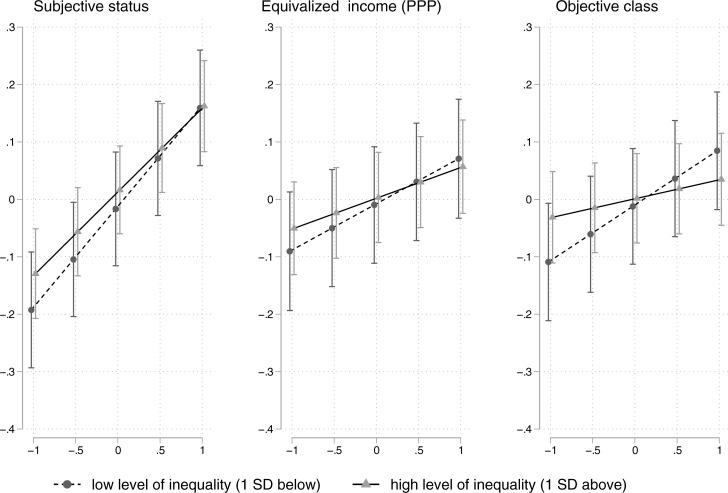
Predicted values of entitlement derived from the models in Table 3. Estimates were calculated for five equally spaced values of our respective class measure (ranging from 1 SD below to 1 SD above average) and for high and low levels of inequality (±1 SD respectively).

Fig 1 allows for a visual inspection of the results shown in Table 3. Although predicted probabilities at especially high and low levels of inequality do not differ on average (as can be seen by the overlapping confidence intervals), individual levels of entitlement converge with an increase in status, income and class. The latter is of particular interest, as we can see that in societies with highly unequal incomes individuals of different objective classes do hardly differ in their levels of entitlement.

## Second inquiry–engaging in volunteer work

Next, we turn our attention towards the question of whether higher levels of inequality cause high-class individuals to act less prosocial. To this end we use the same dataset as before, which also contains a question asking respondents whether they got ‘involved in work for [a] voluntary or charitable organizations’ in the past 12 months. Although this item might (as any survey data) be prone to over-reporting, we still consider it a sufficient proxy for altruistic behavior and thus a suitable dependent variable [19].

First, we conduct several separate multilevel logistic regressions (using Stata’s melogit-ado) of volunteering on subjective status, equalized income as a purchasing power parity, and our composite measure of objective class (Table 4). In all three cases we find the respective aspect of social class to be a highly significant and positive predictor for the probability to engage in voluntary work. To facilitate an easier understanding of the results of the non-linear models, we present regression results in the form of average marginal effects (AMEs), which reflect the average (additive) effect of an independent variable as the mean of the marginal effects over all observations. AMEs thus correspond to the change of probability for the occurrence of our investigated event (volunteering) on the condition of a change of the independent variable by one unit. Thus, an increase in subjective status by one standard deviation corresponds with an increase of 6% in the probability to engage in voluntary work.

**Table 4:** AMEs for the multilevel models regressing volunteering on class measures.

	Subjective status		Equivalized income (PPP)		Objective class	
	AME	SE	AME	SE	AME	SE
Subjective status	0.06***	(0.00)				
Equivalized income (PPP)			0.03***	(0.00)		
Objective class					0.07***	(0.00)
n	20715		20715		20715	

* p < 0.05

** p < 0.01

*** p < 0.001

Following this we calculate the joint models with each of the three class measures respectively, our measure of inequality as a second-level predictor, and their cross-level interaction. We find, as can be seen in Table 5, that the estimates for the direct effects of our class measures stay virtually the same, while regional inequality shows no effect on the respondent’s willingness to volunteer. Further, we find a significant, albeit moderate, positive interaction between inequality and the respondent’s income as well their objective class respectively. This means, that individuals of higher social class (1 SD above average) or higher equivalized income (1 SD above average) living in societies with a high level of inequality (1 SD above average) have each a one percent higher probability to engage in voluntary work than those high-class or high–income individuals living in societies with an average level of inequality.

**Table 5:** AMEs for the multilevel models regressing volunteering on class measures and regional levels of inequality.

	Subjective status		Equivalized income (PPP)		Objective class	
	AME	SE	AME	SE	AME	SE
Inequality	0.01	(0.01)	0.01	(0.01)	0.01	(0.01)
Subjective status	0.06***	(0.00)				
Subjective status * Inequality	-0.00	(0.00)				
Equivalized income (PPP)			0.03***	(0.00)		
Equivalized income (PPP) * Inequality			0.01***	(0.00)		
Objective class					0.06***	(0.00)
Objective class * Inequality					0.01***	(0.00)
n	20715		20715		20715	

* p < 0.05

** p < 0.01

*** p < 0.001

Again, we repeat our analyses using the full set of control variables proposed by Côté et al. to check for possible confounding of our findings. As can be seen in Table 6, our results do not change substantially: Even after controlling for basic sociodemographic (which with the exception of sex do all influence the decision to volunteer) and regional characteristics we only see a minor change of the effect of social class and none for their interaction with inequality.

**Table 6:** AMEs for the multilevel models regressing volunteering on class measures and regional levels of inequality–controlling for additional individual- and regional-level variables.

	Subjective status		Equivalized income (PPP)		Objective class	
	AME	SE	AME	SE	AME	SE
Inequality	0.02	(0.01)	0.01	(0.01)	0.01	(0.01)
Subjective status	0.05***	(0.00)				
Subjective status * Inequality	-0.00	(0.00)				
Equivalized income (PPP)			0.02***	(0.00)		
Equivalized income (PPP) * Inequality			0.01***	(0.00)		
Objective class					0.06***	(0.00)
Objective class * Inequality					0.01**	(0.00)
Age	-0.04***	(0.00)	-0.04***	(0.00)	-0.04***	(0.00)
Gender	-0.01	(0.01)	-0.01	(0.01)	-0.01	(0.01)
Minority	-0.06***	(0.02)	-0.07***	(0.02)	-0.06***	(0.02)
Paid work	0.04***	(0.01)	0.04***	(0.01)	0.01	(0.01)
Married	0.04***	(0.01)	0.05***	(0.01)	0.04***	(0.01)
Religiosity	0.06***	(0.00)	0.07***	(0.00)	0.07***	(0.00)
Conservatism	-0.01**	(0.00)	-0.00	(0.00)	-0.00	(0.00)
Average income (PPP)	0.03*	(0.01)	0.03	(0.01)	0.02	(0.01)
Population size	0.01	(0.01)	0.01	(0.01)	0.01	(0.01)
Percentage urban	-0.00	(0.01)	-0.00	(0.01)	-0.00	(0.01)
Age diversity	0.01	(0.01)	0.01	(0.01)	0.01	(0.01)
Sex diversity	0.04***	(0.01)	0.04***	(0.01)	0.04***	(0.01)
Origin diversity	0.02	(0.01)	0.02	(0.01)	0.02*	(0.01)
n	20715		20715		20715	

* p < 0.05

** p < 0.01

*** p < 0.001

**Fig 2:**
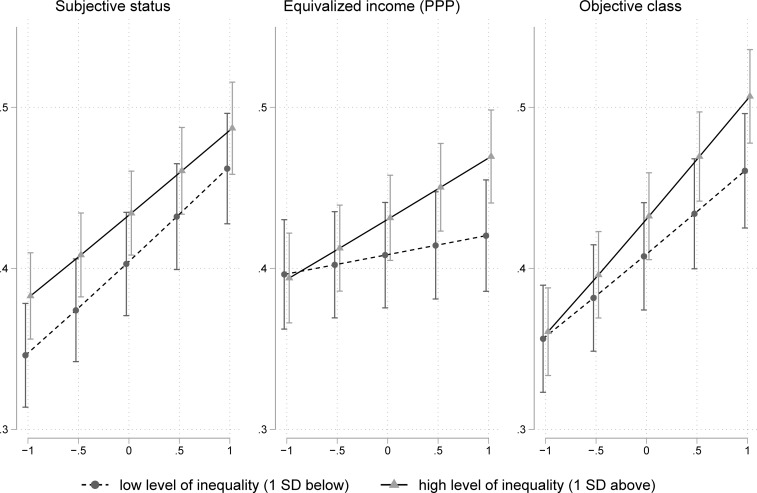
Predicted probabilities of volunteering derived from the models in Table 6.

For a better understanding and since interaction terms in logistic regression models can be misleading, a visual inspection of the class-inequality-interaction seems to be warranted [20]. We therefore calculate predicted probabilities for volunteer engagement on five levels of our class variables at a comparatively high and low level of inequality respectively (1 SD above and below average), which can be seen in Fig 2. While in both cases the probability to work voluntarily increases with a rising social class, this slope is steeper for the objective class in societies with higher levels of inequality, i.e. under conditions of severe inequality, the behavior of high- and low-class subjects differ more sharply than under conditions of mild inequality.

Although volunteering, i.e. the donation of time and labor, can in principle be considered as an act of prosocial behavior, it might be motivated by various reasons. Several organizations for example produce club goods (e.g. sports or social clubs), which would give members an incentive to participate besides pure altruism, while in some cases working (voluntarily) for an organization might even be strongly encouraged and thus correlated with sub-dimensions of the objective class (e.g. occupation in case of professional associations). For this reason, we conduct a third investigation using data from the first wave of the ESS [21].

## Third inquiry–donating money for humanitarian causes

Here respondents were confronted with a matrix question describing various voluntary organizations and asking respondents to indicate whether they donated money to that kind of organization within the past 12 months. This allows us to single out donations to organizations ‘for humanitarian aid, human rights, minorities, or immigrants’. Since it is a reasonable assumption that donations to one of these causes do not promise any direct form of reciprocity, it allows us to effectively eliminate possible conflicts of motivation. While our data preparation and analyses follows the same scheme as described above, we are not able to procure Gini coefficients corresponding with the specific survey year, instead using those from a time period of roughly eight years later (see Measures). Further, the first ESS wave (2002) contains unfortunately no measure for subjective social status. After case-wise deletion of observations with missing values, our final dataset contains 18118 observations clustered in 102 regions across 14 countries.

Again, we set out with a simple multilevel logistic regression of donation on respondent’s equivalized income and objective class. As shown in Table 7, both class measures influence the propensity to donate money positively: People of higher income or objective class (1 SD above average) show a 2%, respective 5%, higher probability to donate money to humanitarian causes than people of average income or class.

**Table 7:** AMEs for the multilevel models regressing donating on class measures.

	Equivalized income (PPP)		Objective class	
	AME	SE	AME	SE
Equivalized income (PPP)	0.02***	(0.00)		
Objective class			0.05***	(0.00)
n	18118		18118	

* p < 0.05

** p < 0.01

*** p < 0.001

Adding inequality and respective interaction terms (Table 8) yields no change in the size and direction of our main effects of income and class. Yet in contrast to our analyses of volunteering, we find a comparatively strong and negative effect of inequality: Thus, an increase in income inequality reduces the prevalence of humanitarian donations. While we find no interaction effect between inequality and income, we can see that the effect of our composite measure of objective class is in fact positively moderated by inequality.

**Table 8:** AMEs for the multilevel models regressing donating on class measures and regional levels of inequality.

	Equivalized income (PPP)		Objective class	
	AME	SE	AME	SE
Inequality	-0.04***	(0.01)	-0.05***	(0.01)
Equivalized income (PPP)	0.02***	(0.00)		
Equivalized income (PPP) * Inequality	0.00	(0.00)		
Objective class			0.05***	(0.00)
Objective class * Inequality			0.01**	(0.00)
n	18118		18118	

* p < 0.05

** p < 0.01

*** p < 0.001

Finally, we repeat our analyses with our full set of individual and regional level control variables, which again do not alter our results in any substantial way. The probability to donate money to humanitarian causes, as can be seen in Table 9, depends on the individual’s income as well as the regional level of inequality, although there is no indication of a moderation of the former by the latter. The propensity to donate increases with higher income (on average 2% per 1 SD increase), regardless of the local level of inequality. Our regression on objective class yields similar results, with the exception of positive and significant although moderate interaction effect. While high-class individuals are more likely to donate to humanitarian causes than individuals of lower class, this discrepancy in fact widens under conditions of higher inequality.

**Table 9:** AMEs for the multilevel models regressing donating on class measures and regional levels of inequality–controlling for additional individual- and regional-level variables.

	Equivalized income (PPP)		Objective class	
	AME	SE	AME	SE
Inequality	-0.04***	(0.01)	-0.05***	(0.01)
Equivalized income (PPP)	0.02***	(0.00)		
Equivalized income (PPP) * Inequality	0.00	(0.00)		
Objective class			0.06***	(0.00)
Objective class * Inequality			0.01**	(0.00)
Age	0.00	(0.00)	0.00	(0.00)
Gender	0.04***	(0.01)	0.04***	(0.01)
Minority	0.01	(0.01)	0.01	(0.01)
Paid work	0.03***	(0.01)	0.01	(0.01)
Married	0.02***	(0.01)	0.02**	(0.01)
Religiosity	0.02***	(0.00)	0.02***	(0.00)
Conservatism	-0.02***	(0.00)	-0.02***	(0.00)
Average income (PPP)	0.05***	(0.01)	0.05***	(0.01)
Population size	-0.03*	(0.01)	-0.03*	(0.01)
Percentage urban	0.01	(0.01)	0.00	(0.01)
Age diversity	0.01	(0.01)	0.01	(0.01)
Sex diversity	0.00	(0.01)	0.00	(0.01)
Origin diversity	0.00	(0.01)	0.00	(0.01)
n	18118		18118	

* p < 0.05

** p < 0.01

*** p < 0.001

**Fig 3:**
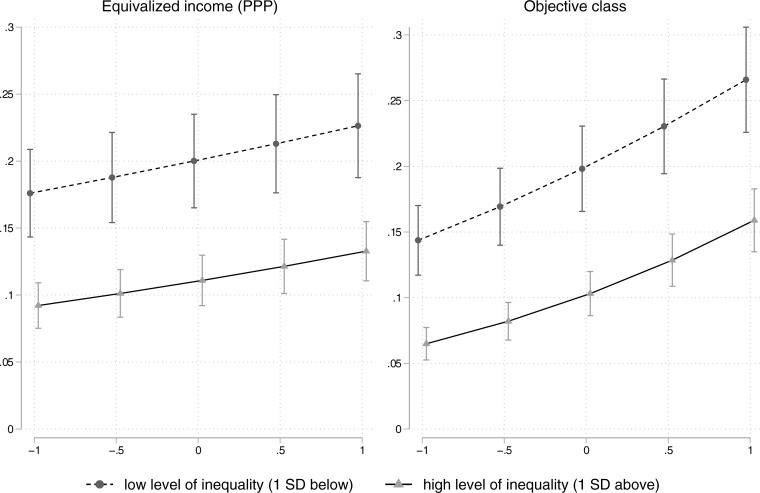
Predicted probabilities of donating derived from the models presented in Table 9.

Although the interaction effect of inequality and objective class seems substantial (having one-sixth of the effect size of class) a visual inspection puts this finding into perspective. While both main effects can be easily identified in Fig 3, an interaction is hard to recognize.

## Discussion

The scientific community is currently divided on the relation between social class and prosocial behavior. By now, both schools of thought have ample evidence to show in support for their respective thesis, ranging from (small-scale) experiments to large-scale surveys. We report results from two cross-national surveys [9,21], showing that individuals of higher social class are more likely to donate money to humanitarian causes and engage in volunteer work than individuals of lower social class. Alternative specifications, focusing on a single aspect of social class (i.e. income and subjective status), produce consistent findings and point in the same direction. As such, our results add to the growing literature that considers social class to be a strong positive predictor of at least some forms of prosocial behavior. Further, we put Côté et al.’s intriguing idea to an empirical test. That is, we test whether taking the regional level of inequality into account allows to reconcile the aforementioned conflicting evidences. It was argued that inequality moderates the effect of class on individual’s sense of entitlement as well as the willingness to act prosocial. We test both claims, but are unable to confirm either. Instead of a positive interaction with respect to sense of entitlement and a negative interaction regarding prosocial behavior, our results, while not fully consistent, point to the respective opposite. While high-class subjects–irrespective of the degree of inequality–feel more entitled yet act more prosocial than lower-class subjects, high levels of inequality tend towards encouraging them to be more modest and act even more prosocial. Controlling for additional first- or second-level variables as proposed by Côté et al. does not change our findings in any substantial way, suggesting an overall robustness.

Of course, our results are subject to various limitations which must be considered while drawing conclusions. First, both forms of prosocial behavior under consideration, i.e. monetary donations to humanitarian causes and volunteering for charitable organizations, as well as self-enhancement, which we interpret as a proxy of entitlement, but which can also be considered as some form of prosocial orientation, were not directly observed but self-reported by the respondents and hence prone to over-reporting. While this is certainly a drawback, it must be stressed that over-reporting only biases our main finding if high-class subjects engage in more over-reporting than low-class subjects. Even if the latter assumption would hold true, it seems implausible that it accounts for the considerable effect sizes of social class observed in our study. Further, it should be noted, that due to the dichotomous nature of our measure, the variance in the extent of charitable giving and volunteering explained by social class could not be investigated.

Second, it proved to be quite difficult to obtain data on important variables on the regional level and as a consequence some regional variables are not perfectly matched to the data on the individual level in terms of timing. For instance, we were unable to acquire regional estimates for the median income, relying instead on an average. Most importantly, in the third inquiry we had to rely on Gini coefficients from around 2010 whereas the individual data stem from 2002. To remedy these problems regarding the availability and timing of data and bolster the reliability of our findings, we performed our analyses with respect to two different measures of prosocial behavior.

The third major limitation of the current study is most informative regarding directions of future research. As already pointed out almost all studies which find a negative effect of social class on prosocial behavior work with data from the US, whereas almost all studies which rely on data from the EU find a positive effect. Against this background, Côté et al. reasoned that economic inequality might be the critical moderating context variable that causes the reversal of the class-effect. Our study failed to support this reasoning. However, since we only relied on data from the EU, the question, which context variable moderates the effect of class on prosocial behavior such that the inconsistent evidences reported in the literature occur, remains open. We believe that cultural differences between the US and the EU might be more important than differences in economic inequality. For instance, quantitative cultural research indicates inhabitants of the US are much more individualistic oriented than EU citizens [22,23]. Since this cultural trait is known to impact prosocial behavior [24], individualism might moderate the effect of social class on prosociality and potentially explain the conflicting evidences from the US and the EU. Further, future research on the interplay of social class and prosocial behavior should address whether the supposed mechanisms vary for different social classes. While for example favorable downward comparisons of high-class actors might induce a sense of entitlement, unfavorable upward comparisons of low- to middle-class actors could induce relative deprivation, which would also affect prosocial orientations and behaviors [25].

## Measures

### Entitlement

All ESS waves so far contained an item battery to derive human value dispositions as proposed by Schwartz [12]. To this end, interviewer read respondents a list of description of a fictive person, asking them to what degree each person resembles them or not (1 –very much like me; 6—not like me at all). After reversal of the response categories our four items—describing power (important ‘to be rich, […] have lots of money and expensive things’; ‘to get respect, […] people do what she/he says’) and achievement values (important ‘to show abilities, […] wants to be admired’; ‘very successful, […] recognize achievements’)—show a scale reliability of α = .73. We derive a joint measure of entitlement by principal component analysis (PCA), which returns a single factor solution with an eigenvalue of 2.22, an explained variance of 55.5% and a Kaiser-Meyer-Olkin (KMO) value of 0.75.

### Volunteering

The original question read: ‘In the past 12 months, how often did you get involved in work for voluntary or charitable organisations?’ with answers ranging from 1 ‘at least once a week’ to 6 ‘never’. We recode the item into a binary variable with 0 equal to never and 1 all else—42.7% of all respondents stated to have engaged in voluntary work at least once in the past year.

### Donating

The item was part of matrix question which was introduced as follows: ‘For each of the voluntary organisations I will now mention, please […] tell me whether any of these things apply’. Our final variable is coded as 1 if respondents answered that they had donated money to ‘an organisation for humanitarian aid, human rights, minorities, or immigrants’ and 0 if not—16.5% of all respondents donated money to a humanitarian organization in the past year.

### Income inequality

**Fig 4:**
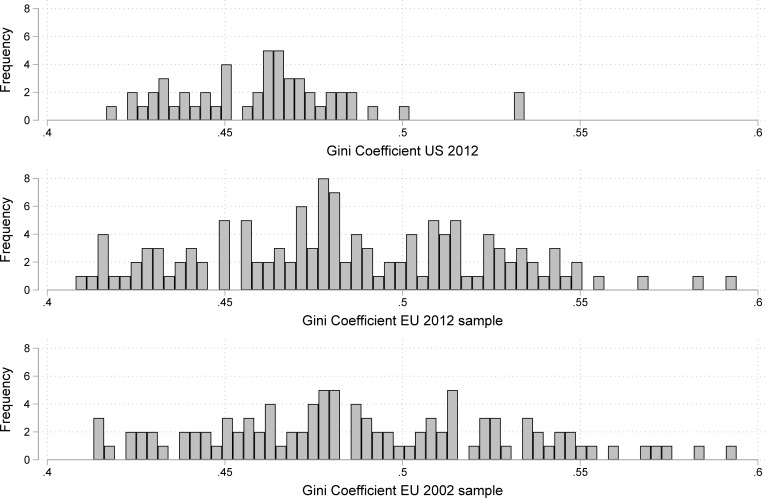
Comparison of Gini coefficients for US states in 2012 and NUTS regions.

Second-level standard errors in multilevel regression models can be biased by an insufficient sample size at that level [26]. While Gini coefficients of income inequality are available for almost all countries and years of the ESS, the number of countries per wave is far below the recommended threshold (n> = 50), which is why we drafted our measure of inequality from the OECD database of *Regional income distribution and poverty* [11]. Relying on various national sources (administrative data as well as household surveys), the OECD compiled national data on the first level of administrative subdivision for each country (mainly corresponding to NUTS regions), which refer in most cases to a single year around 2010 (for a detailed overview see: https://www.oecd.org/cfe/regional-policy/Regional-Income-distribution-database.pdf). After case-wise deletion of missing values and before standardizing, the *Gini coefficient for the equivalized household disposable income* range in the used cases of the first ESS wave from .413 to .594 (M = .473, SD = .047) and for the cases of the sixth wave from .408 to .594 (M = .47, SD = .04). Fig 4 allows a visual comparison of the levels of inequality reported by Côté et al. for the US states in 2012 and those of the NUTS regions used in our analyses.

### Subjective status

In the sixth ESS wave respondents were presented a variant of the MacArthur scale, asking them where they would place themselves on a scale ranging from 0 (‘Bottom of our society’) to 10 (‘Top of our society’; M = 5.8, SD = 1.7).

### Education

Respondents education in the ESS is reported using the seven tier International Standard Classification of Education (ISCED), reaching from ‘less than lower secondary’ to ‘higher tertiary education’. For 41.8% of the used cases in the first ESS wave individual educational attainments was not harmonized with the ISCED coding. These cases were reassigned to ISCED through the number of years of full-time education, with less than four years corresponding to ISCED-0 (coded as 1) and more than 18 years to ISCED-6 (coded as 7) respectively (ESS1: M = 3.82, SD = 1.67; ESS6: M = 3.98, SD = 1.82).

### Occupational prestige

Occupational prestige is measured using Treiman’s Standard International Occupational Prestige Scale (SIOPS) [27], which can be derived from the International Standard Classification of Occupation (ISCO) via correspondence tables available from the Homepage of Harry Ganzeboom (http://www.harryganzeboom.nl). SIOPS values based on the ISCO88 standard in the first ESS wave range from 6 (‘Hunters & Trappers’) to 78 (Medical Doctors & University teachers; M = 41.5, SD = 13.4) and those based on the newer ISCO08 standard in the sixth wave range from 5 (‘Subsidiary farmers’) to 78 (M = 42.2, SD = 14).

### Income

Our measure of income is based on the household’s total net income reported in the ESS. For the first three waves respondents picked an income bracket from a show card, containing 12 categories, which were equal for all participating countries (and reported in the dataset in Euro), yet were shown to the respondent in their local currency. Starting from the fourth wave, income was reported using a 10-tier spacing based on the local currency, which was customized by the national coordinators and which produced diverging categories per country and year (allowing a better reflection of the national income distribution). Therefore, we had to take several steps to obtain a metric and harmonized income variable. First, we defined midpoints for each income bracket and estimated a corresponding value for the top-most category via extrapolation [28]. Then we derived household weights, which applied a factor of one to every first adult per household, 0.5 to every additional adult (or unknown) household member and 0.3 to every household member below the age of 14. The household’s net income divided by this score produced the equivalized household’s net income. Next, we used yearly averaged historical exchange rates to convert reported Euro-values back into their (partly discontinued) local currencies [29]. Finally, we apply an exchange rate to purchasing power parity (PPP) in US Dollar as reported by the OECD [17], resulting in our final indicator which reflects a common metric of disposable income for all respondents, irrespective of their place of dwelling (ESS1: M = 1529, SD = 1198; ESS6: M = 1837, SD = 1109).

### Objective class

To obtain a proxy for the objective social class we first standardized our measures of education, occupational prestige and income globally, i.e. over all observations, regions and countries. Following this we calculate a simple average, which we then again standardize globally.

### Individual-level control variables

We control for several individual variables that could correlate with our dependent variables as well as some or all status measures: age (five-year intervals), sex (0 = male, 1 = female), marital status (0 = not married, 1 = married), belonging to a minority (0 = no minority, 1 = minority), doing paid work in the past seven days (0 = no paid work; 1 = doing paid work), religiosity and conservative ideology. The last two items are a regular part of the standard ESS questionnaire, with the former asking respondents how religious they are on a scale of 0 (‘not religious at all’) to 10 (‘very religious’), and the latter to place themselves on a political spectrum ranging from left (0) to right (10; ESS1: religiosity: M = 4.8, SD = 2.9; conservatism: M = 5, SD = 2.1; ESS6: religiosity: M = 4.3, SD = 3.1; conservatism: M = 5.1, SD = 2.3).

### Regional-level control variables

We select our second-level control variables in accordance with the analyses of Cote et al., although we have to make concessions towards their limited availability on a regional level. In some cases, namely Norway and the Netherlands, we therefore aggregate NUTS regions back to state- or country-level, which result in large second-level clusters and a respective population size between 83845 and 18052092 per region (ESS1: M = 5033367, SD = 4848049; ESS6: M = 4839916, SD = 4556501). Due to changes in the NUTS classification we drop regions of the first ESS wave that cannot be aligned with later data points. Further, we fill gaps in the time-series data of Eurostat by ‘raking’ and replacing missing values with their next recent observation [11].

We control for the regional income level by using an estimate of the average Euro per inhabitant per year reported in a purchasing power standard based on final consumption by Eurostat (ESS1: M = 15877, SD = 4398; ESS6: M = 18106, SD = 5021). For our measures of urbanity, we divide the number of households located in designated city areas by the total number of households per region (ESS1: M = .49, SD = .25; ESS6: M = .38, SD = .23). As diversity measures for sex, age and ethnicity we calculate the generalized variance ($1-p_{k}^{2}$, where *k* identifies the category and *p* its relative proportion), also known as Blau’s or Simpson-Index, which gives the probability that two units from a random draw are part of the same category. Age is grouped into 20 categories, covering five years each. As a proxy for ethnicity we use the legal status, differentiating citizens and non-citizens (incl. citizens of other European countries; ESS1: age: M = .94, SD = .002; sex: M = .50, SD = .0003; citizens: M = .11, SD = .07; ESS6: age: M = .94, SD = .002; sex: M = .50, SD = .0003; citizens: M = .10, SD = .07).
